# Gallotannin-rich *Caesalpinia spinosa* fraction decreases the primary tumor and factors associated with poor prognosis in a murine breast cancer model

**DOI:** 10.1186/1472-6882-13-74

**Published:** 2013-04-03

**Authors:** Claudia Urueña, Juan Mancipe, John Hernandez, Diana Castañeda, Luis Pombo, Alejandra Gomez, Alexzander Asea, Susana Fiorentino

**Affiliations:** 1 Grupo de Inmunobiología y Biología Celular. Departamento de Microbiología. Facultad de Ciencias, Pontificia Universidad Javeriana, Carrera 7 N. 43-82 Building 52, Office 608, Bogotá, Colombia; 2 Grupo de Farmacología Vegetal, Fundación Universitaria Juan N. Corpas, Carrera 111 #159 A 61, Bogotá, Colombia; 3 Department of Microbiology, Biochemistry & Immunology, Morehouse School of Medicine, 720 Westview Avenue SW, Atlanta, GA, 30310, USA

**Keywords:** *Caesalpinia spinosa*, Breast cancer, Apoptosis, 4T1, IL-6, Metastasis

## Abstract

**Background:**

Several treatment alternatives are available for primary breast cancer, although those for metastatic disease or inflammation associated with tumor progression are ineffective. Therefore, there is a great need for new therapeutic alternatives capable of generating an immune response against residual tumor cells, thus contributing to eradication of micrometastases and cancer stem cells. The use of complex natural products is an excellent therapeutic alternative widely used by Chinese, Hindu, Egyptian, and ancestral Latin-American Indian populations.

**Methods:**

The present study evaluated cytotoxic, antitumor, and tumor progression activities of a gallotannin-rich fraction derived from *Caesalpinia spinosa* (P2Et). The parameters evaluated *in vitro* were mitochondrial membrane depolarization, phosphatidylserine externalization, caspase 3 activation, DNA fragmentation, and clonogenic activity. The parameters evaluated *in vivo* were tumor growth, leukocyte number, metastatic cell number, and cytokine production by flow cytometry.

**Results:**

The *in vitro* results showed that the P2Et fraction induced apoptosis with mitochondrial membrane potential loss, phosphatidylserine externalization, caspase 3 activation, DNA fragmentation, and decreased clonogenic capacity of 4T1 cells. *In vivo*, the P2Et fraction induced primary tumor reduction in terms of diameter and weight in BALB/c mice transplanted with 4T1 cells and decreased numbers of metastatic cells, mainly in the spleen. Furthermore, decreases in the number of peripheral blood leukocytes (leukemoid reaction) and interleukin 6 (IL-6) serum levels were found, which are events associated with a poor prognosis. The P2Et fraction exerts its activity on the primary tumor, reduces cell migration to distant organs, and decreases IL-6 serum levels, implying tumor microenvironment mechanisms.

**Conclusions:**

Overall, the P2Et fraction lessens risk factors associated with tumor progression and diminishes primary tumor size, showing good potential for use as an adjuvant in breast cancer ER(+) treatment.

## Background

One of the most common cancers in women worldwide is breast cancer. Its estimated incidence is 23% (1.38 million) new cases per year, and it comprised 14% (458,000) of all cancer deaths in 2008 [1]. According to the Colombian Cancer Institute, there were 551 new cases localized at the primary site in 2009, corresponding to 10% of all newly registered cancer cases [2]. There are several therapeutic alternatives for breast cancer, including surgery, hormone therapy, chemotherapy, and radiotherapy. Nonetheless, these therapies are ineffective for metastatic disease [3]. Therefore, there is a great need for new therapeutic alternatives capable of generating an immune response against residual tumor cells, thus contributing to eradication of micrometastases and cancer stem cells [4]. The use of complex natural products is an excellent therapeutic alternative widely used by Chinese, Hindu, Egyptian, and ancestral Latin-American Indian populations [5]. Biologically active compounds, such as hydrolyzable or condensed tannins [6], have high therapeutic potential in cancer treatment. Green tea-derived condensed tannins, for example, have been extensively studied. Their activities include tumor growth inhibition, decreased cell proliferation, increased apoptosis, and angiogenic suppression [7]. On the contrary, studies on the biological activity of hydrolyzable tannins are limited. Nevertheless, reports have shown that the tannin fraction derived from *Eugenia jambos* L. (Myrtaceae) is active against HL60 leukemia cells, inducing apoptosis and DNA fragmentation [8]. In addition, a gallotannin-rich fruit extract from *Terminalia chebula* induces apoptosis in the murine and human breast cancer cells S115 and MCF-7, respectively [9].

Soluble mediators may be exchanged between tumor cells and their microenvironment, affecting tumor growth and promoting metastases. Such mediators may be secreted as a result of the interaction between the innate and/or adaptive immune system with tumor cells, or produced by the tumor cell to modulate its surroundings for its own benefit [10]. Several reports indicate that high IL-6 serum levels in patients with breast carcinoma indicate a poor prognosis due to correlation with metastases in patients with untreated breast cancer [11].

Our group recently reported that a *Caesalpinia spinosa*-derived fraction enriched in hydrolyzable tannins induces a cytotoxic effect on leukemia cells (K562), apoptosis with mitochondria membrane potential (MMP) loss, DNA fragmentation, and a decrease in clonogenic capacity [12]. Additional reports on the same plant have shown that hydrolyzable tannins such as tannic acid (TA) lower the biochemical marker levels associated with skin cancer and delay the development of tumors in mouse skin [13,14]. However, reports on breast cancer have not been linked with these plant-derived tannins.

The murine breast adenocarcinoma cell line 4T1 is poorly immunogenic and highly invasive and expresses the estrogen receptor (ER+). When orthotopically transplanted into BALB/c mice, 4T1 cells are able to reproduce stage IV human breast cancer pathology [15]. Primary tumor metastasizes spontaneously to the lung, liver, brain, and bone marrow [16], making this an excellent model to study metastatic breast cancer pathophysiology and develop effective therapies.

We evaluated the effects of a *C. spinosa* plant fraction on 4T1 cells *in vitro* and *in vivo*. The fraction induced tumor cell apoptosis *in vitro*, an activity related to IL-6 secretion by the tumor cells. *In vivo*, it inhibited tumor growth, reduced metastatic cell numbers, and reduced peripheral blood leukocytes. Finally, IL-6 serum levels in fraction-treated mice were lower than those in untreated mice.

## Methods

### Plant material, extraction and purification

*Caesalpinia spinosa* pods were collected in Villa de Leyva, Boyacá, Colombia in March 2007 and identified by Luis Carlos Jiménez from the Colombian National Herbarium (voucher specimen number COL 523714). Authorization to collect the vegetal material was given by “Ministerio de Medio Ambiente y Desarrollo sostenible de Colombia” (Nº 5 from 10 may 2011). P2Et fraction was purified and characterized as previously described [12].

### Cell culture conditions

The murine mammary tumor cell line 4T1 (a gift from Alexzander Asea of Texas A&M Health Science Center College of Medicine, Temple, TX, USA) was cultured in RPMI-1640 (Eurobio, Toulouse, France) supplemented with heat-inactivated fetal calf serum (10%) (Eurobio), 2 mM L-glutamine, 100 U/mL penicillin, 100 μg/mL streptomycin, 0.01 M Hepes buffer, and 1 mM sodium pyruvate (Eurobio) and incubated in a humidified environment at 37°C and 5% CO_2_. Tumor cells were proven *Mycoplasma*-free with a MycoProbe Mycoplasma Detection Kit (R&D Systems, Minneapolis, MN) and maintained with ciprofloxacin (0.5 μg/mL). Cells were grown until 75% confluence, removed with trypsin/EDTA 1X (Eurobio), washed with phosphate-buffered saline (PBS) (154 mM, pH 7.2), and suspended in supplemented RPMI-1640. Human peripheral blood mononuclear cells (PBMC) and fibroblasts obtained from normal healthy donors (with informed consent) were used as normal cells. Normal cells were maintained as previously reported [17]. This project was approved by the ethics committee (founded in 2002) of the Science Faculty at a meeting on August 21, 2007.

### *In vitro* cytotoxicity assays

P2Et fraction and doxorubicin cytotoxic effects on normal and tumor cells were evaluated using trypan blue and methylthiazol tetrazolium (MTT) assays (Sigma-Aldrich, Saint Louis, MO) as previously reported [17]. Cell viability was assessed with a trypan blue dye exclusion test. The IC50 (50% inhibition of cell growth) value was calculated using Probit analysis (MINITAB Release 14.1; Minitab Inc. 2003 Statistical Software).

### Evaluation of MMP

Cells (3 × 10^5^) were treated with various concentrations of P2Et fraction (34.1, 17, and 8.5 μg/mL), valinomycin (positive control, 0.1 μg/mL) or ethanol (negative control, 0.02%) for 6, 12, 24, and 48 h. MMP was measured using JC-1 (Sigma-Aldrich) dye as previously reported [17]. The cells were acquired on a FACSAria I (Becton, Dickinson and Company, Franklin Lakes, NJ) and analyzed with FlowJo software (Tree Star Inc., Ashland, OR), which calculated the red/green fluorescence ratios. Duplicate estimations were made, and the average was expressed as mean ± SEM in three independent experiments.

### Annexin V assay

Phosphatidylserine (PS) externalization was assessed by flow cytometry using Annexin V-Alexa Fluor 488 (Molecular Probes, Invitrogen Corp., Carlsbad, CA) and propidium iodide (PI) (Sigma-Aldrich) as previously reported [12]. Briefly, 4T1 cells (3 × 10^5^) were treated with P2Et fraction (34 and 17 μg/mL), doxorubicin (positive control, 0.51 and 0.27 μg/mL), or ethanol (negative control, 0.02%) for 24 and 48 h. After treatment, the cells were dyed with Annexin V and PI, acquired on a FACSAria I (Becton Dickinson), and analyzed with FlowJo software (Tree Star Inc). Assays were performed in triplicate.

### Caspase 3 activity assay

Caspase 3 activity was estimated using a caspase 3 colorimetric assay kit (Sigma-Aldrich). Briefly, cells (2 × 10^5^ cells) were cultured at various concentrations of P2Et fraction, doxorubicin (positive control), or ethanol (negative control, 0.02%) for 48 h. Caspase 3 activity was estimated following the manufacturer’s instructions. The increase in caspase 3 activity was calculated from a calibration curve prepared with pNA standards using the following formula: Activity, μmol pNA/min/mL = [(μmol pNA × d)/(t × v)], where d = dilution factor, t = reaction time in min, and v = volume of sample in milliliters.

### Analysis of DNA fragmentation

Cells (3 × 10^5^) were treated with different concentrations of P2Et fraction (34.1 and 17 μg/mL), doxorubicin (positive control, 0.27 μg/mL) or ethanol (negative control, 0.02%) for 48 h. DAPI (4',6-diamidino-2-phenylindole; Sigma-Aldrich)-stained cells were monitored under a confocal microscope as previously reported [17]. Slides were mounted using a ProLong Antifade Kit (Molecular Probes), and cells were analyzed under a fluorescence confocal microscope (FluoView 1000; Olympus, Japan).

### Clonogenic assays

The clonogenic assays were performed as previously described [17]. Briefly, 4T1 cells (2.5 × 10^5^ cells) were plated (96-well plates) and treated with P2Et fraction (34.1, 17, and 3.4 μg/mL), doxorubicin (0.51, 0.25, and 0.17 μg/mL), or ethanol (negative control, 0.02%) and incubated for 6 h in a humidified environment at 37°C and 5% CO_2_. After treatment, cells were re-plated onto 0.5% agar dishes (60 mm; 20,000 cells/dish), incubated for 14 d (37°C and 5% CO_2_), and stained with violet crystal (0.4% in ethanol). Cell colonies with >50 cells were counted. Treatments were performed in triplicate, and results are expressed as mean ± SEM.

### Cytokine secretion assessment

Cytokine secretion was assessed by flow cytometry using a BD Cytometric Bead Array (CBA) Mouse Inflammation Kit (Becton Dickinson). Briefly, 4T1 cells (2 × 10^6^ cells) were cultured with different concentrations of P2Et fraction (34, 17, and 3.4 μg/mL), doxorubicin (0.5, 0.27, and 0.05 μg/mL), or ethanol (negative control, 0.02%) for 6, 12, 24, and 48 h. Secreted cytokines were acquired on a FACSAria II (Becton Dickinson) and analyzed with FCAP Array software (Becton Dickinson). Treatments were performed in triplicate, and results are expressed as mean ± SEM.

### Mice

Female BALB/c mice, 6 to 12 weeks old and purchased from Charles Rivers Laboratories International, Inc. (Wilmington, MA), were housed in our animal research facility following the established protocols of the Ethics Committee of the Science Faculty and National and International Legislation for Live Animal Experimentation (Colombia Republic, Law 84, 1987; Colombia Republic, Resolution 08430, 1993; National Academy of Sciences, 2010). Mice were housed in polyethylene cages with food and water ad libitum, controlled temperature, and a 12-h light/dark cycle. Before treatments, the mice were acclimated for 1 week under standard conditions.

### Acute toxicity evaluation

Female BALB/c mice (6 to 12 weeks of age) weighing approximately 25 g, were divided into groups of five and inoculated intraperitoneally (IP) with 1, 2, 4, and 8 mg/mL of P2Et fraction corresponding to 40, 80, 160 and 320 mg/kg. After 72 h, deceased animals were counted and the lethal dose, 50% (LD50) was calculated with Probit version 14 (Minitab Inc.). To ensure no toxicity animals were treated with two different doses of P2Et fraction (18.7 and 9.3 mg/Kg) which corresponds to 4 times lower doses than LD50.

### Tumor model, treatment protocol, and cytokine detection

4T1 cells (1 × 10^4^) suspended in 100 μL of saline phosphate-buffer were injected into the right mammary fat pad (subcutaneously [SC]) on day 0. After 5 days of inoculation, the mice (7–8 mice/group) were treated with IP injection of P2Et fraction (9.3 or 18.7 mg/kg) or control vehicle twice a week. The leukocyte count was determined weekly using a hemocytometer (ABX SAS Micros 60; Horiba, Madrid, Spain). Blood was obtained from a small incision made to a lateral tail vein and added directly into an EDTA containing vial and then diluted 1:1 with PBS and counted immediately using a Calibrated Cell Counter. Additionally, differential counts were performed using Wright staining. Tumors were measured with vernier calipers twice a week, and tumor volume was calculated using the following formula: tumor volume (mm^3^) = [(width)^2^ × length]/2 [18]. Mice were sacrificed when the primary tumor reached 2000 mm^3^, and lung, spleen, liver, brain, primary tumor, and bone samples were collected for histopathology studies. Tumor weight was determined in a high-sensitivity balance. Serum was collected by cardiac puncture, and cytokine evaluation was performed using a BD CBA Mouse Inflammation Kit (Becton Dickinson). Experiments were performed twice, and the results are expressed as the mean of two independent experiments.

To evaluate the effectiveness of oral treatment with P2Et fraction, animals were randomly assigned in control or treatment groups (7 mice per group). One week prior to tumor cell inoculation (day 0), the treatment group started P2Et fraction consumption (0.306 mg/Kg daily equivalent to dose schedule used in IP administration experiment plus an adjustment for bioavailability) administered in water. 4T1 cells (1 × 10^4^) suspended in 100 μL of PBS were injected into the right mammary fat pad SC on day 7. All animals were weighed once a week and food and water consumption was recorded once a week. To identify and quantitate surface lung metastatic nodules, at the end of the experiment, animals were euthanized and the chest cavity and trachea was exposed through a midline chest incision. The trachea was cannulated with a 30-gauge syringe, and the lungs were slowly insufflated with India Ink (1:15 PBS). The lungs were excised *en bloc* and immersed in Fekete’s solution (EtOH 70%, formaldehyde 10%, glacial acetic acid at a 60:22:1 ratio) to destain the pulmonary nodules. White tumor nodules against a blue lung background were counted by two independent observers.

### Histopathologic analysis

After treatment with P2Et fraction, primary tumors, lung, liver, spleen, brain, and bone tissues were dissected and fixed in 10% formaldehyde (Sigma-Aldrich), embedded in paraffin, cut into 2-μm sections, and stained with hematoxylin and eosin (H&E). Metastatic tumor infiltrations were evaluated for each tissue and counted under a microscope using a micrometric grid and counted in 5 different fields (magnification power, 10× or 40×).

### Statistical analysis

Values are given as mean ± SEM. Data were analyzed by one- and two-way ANOVA, and differences between the control and treated groups were determined using the Bonferroni test. Differences were considered significant at p < 0.05 and determined with GraphPad Prism 5.0 software (La Jolla, CA, USA).

## Results

### P2Et fraction is cytotoxic to 4T1 tumor cells *in vitro*

The cytotoxicity of P2Et fraction on 4T1 cells was evaluated by MTT assay. Tumor cells were treated with P2Et fraction (at different concentrations), gallic acid, and doxorubicin for 48 h. The fraction reduced 4T1 cell viability in a dose-dependent manner with an IC50 of 34.1 μg/mL. Notably, one of the compounds present in P2Et fraction is gallic acid, and gallic acid by itself exhibits less cytotoxicity (96.2 μg/mL) than does the full P2Et fraction. This suggests that compounds other than gallic acid exert this cytotoxic activity [19]. Doxorubicin was used as positive control with an IC50 of 0.5 μg/mL, as expected (Table 1, Additional file 1: Figure S1). To evaluate whether the fraction activity is selective enough for tumor cells, normal fibroblasts and human mononuclear cells were treated with P2Et fraction. The outcome was that P2Et fraction exerted a slight cytotoxic effect on normal cells at high doses where the IC50 was more than 125 μg/mL, as shown in Table 1.

**Table 1:** **IC**_**50 **_**values of P2Et fraction, gallic acid, and doxorubicin in 4T1 and normal cells (gingival fibroblasts and PBMC) estimated using the MTT assay**

	**IC**_**50 **_**values after treatment with the following compounds**		
**Cell Type**	P2Et fraction*	Gallic acid*	Doxorubicin*
4T1	34.1 ± 1.6	96.2 ± 0.0	0.5 ± 0.1
PBMC + PHA	> 125 ± 1.0	ND	2023 ± 0.2
Fibroblasts	> 125 ± 1.0	ND	0.4 ± 0.1

*Concentration (μg/mL) ± SEM.

*ND* = not done.

### P2Et fraction induces apoptosis through the mitochondrial pathway

**Figure 1:**
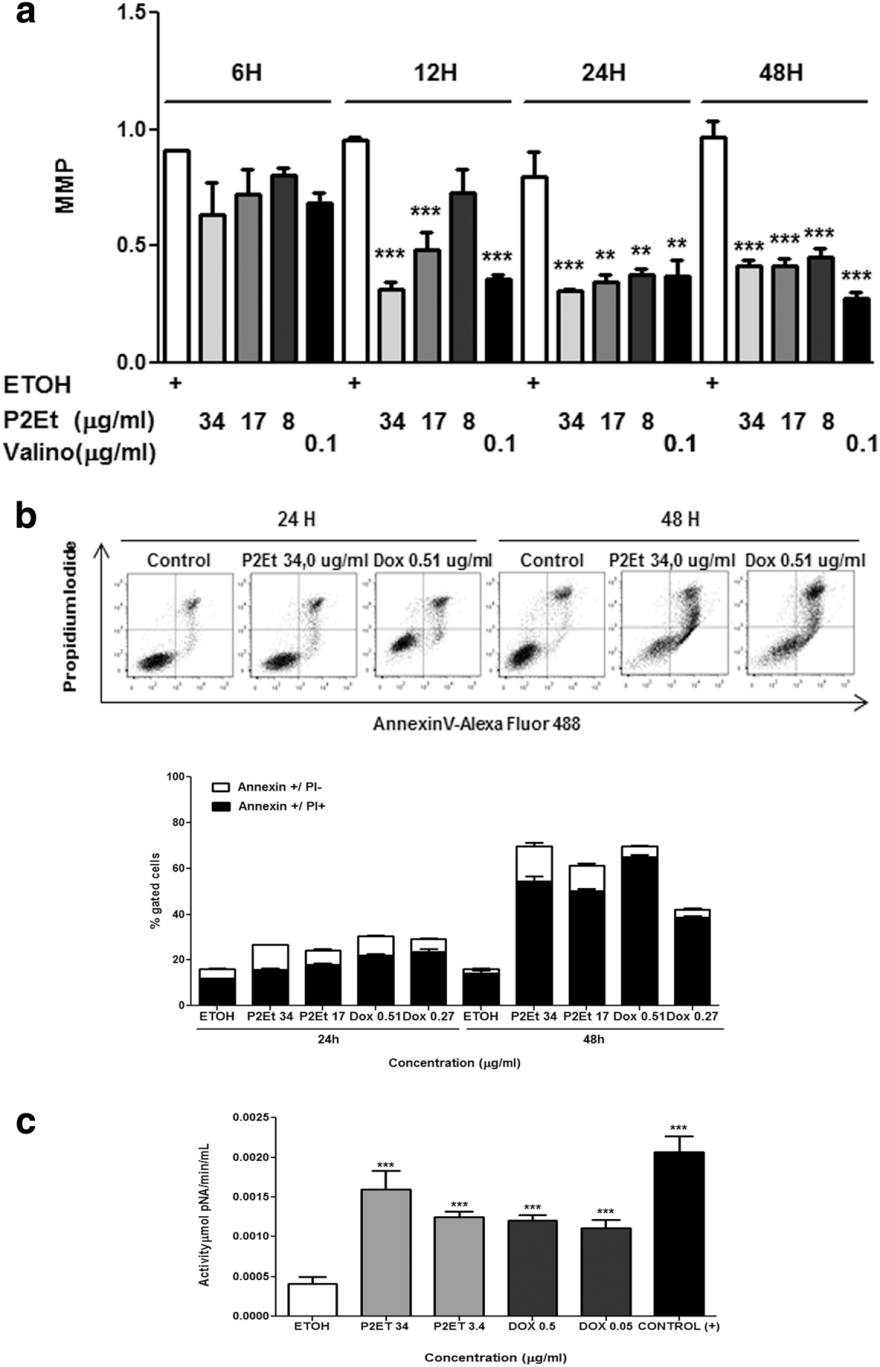
**MMP loss, PS externalization, and activation of caspase 3 in 4T1 cells induced by P2Et fraction.** (**a**) 4T1 cells treated with P2Et fraction for 6, 12, 24, or 48 h, stained with JC-1, and analyzed by flow cytometry. Ethanol (negative control) and valinomycin (positive control) (***p < 0.001). (**b**) PS externalization in 4T1 cells by flow cytometry after treatment for 24 h and 48 h with P2Et fraction, doxorubicin (positive control), or ethanol (negative control). Top panel is a representative dot plot showing the various treatments; bottom is a representative histogram showing percent (%) PS/PI cells after 24 and 48 h of treatment. (**c**) Caspase 3 activity by enzyme-linked immunosorbent assay (ELISA) after cell treatment with P2Et fraction or doxorubicin (positive control) for 48 h (***p <0.001).

**Figure 2:**
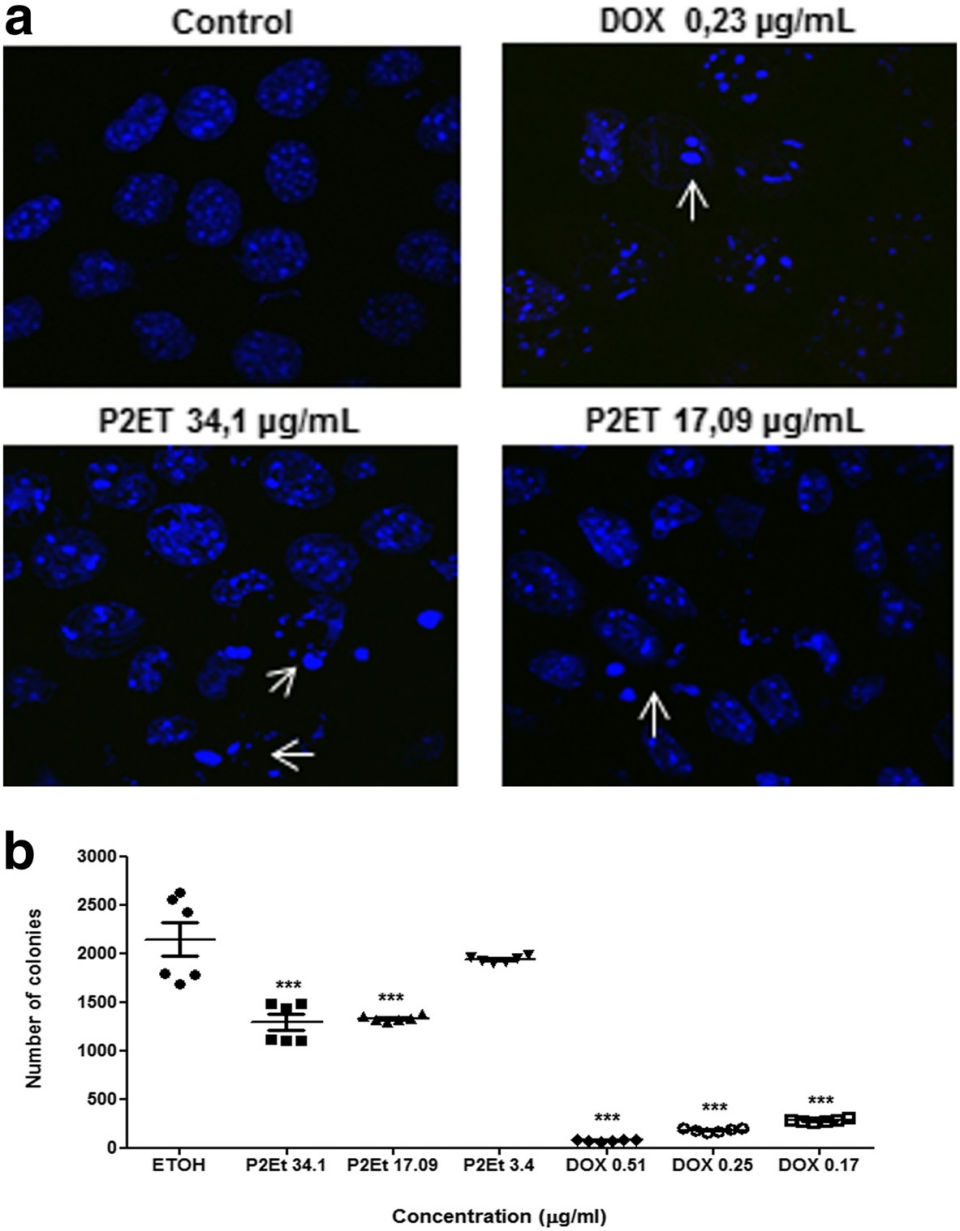
**P2Et fraction decreases 4T1 cell nuclear segmentation and clonogenic activity.** (**a**) 4T1 cells treated with P2Et fraction for 48 h and stained with DAPI. White arrows show apoptotic cells or fragmented nuclei observed under a fluorescence microscope (40×). (**b**) 4T1 cells treated with P2Et fraction, ethanol (negative control), and doxorubicin (positive control). Data represent the mean number of colonies ± SEM of three independent experiments (***p < 0.001) versus control.

Defining the type of tumor cell death induced by cytotoxic or cytostatic agents may give information about the patient’s response. To assess the cell death type induced by P2Et fraction, we estimated loss of MMP, externalization of PS, activation of effector caspases, and DNA fragmentation [20]. 4T1 cells treated with P2Et fraction at different concentrations for 6, 12, 24, and 48 h showed MMP loss in a dose-dependent manner starting at 6 h after treatment until 48 h (Figure 1a). Similarly, PS externalization was evidenced by Annexin V-Alexa Fluor 488 dye, where Annexin V+/PI- cells were early apoptotic cells (Figure 1b). In addition, the late appearance of Annexin V+/PI + cells at 48 h after treatment showed the cell death was apoptotic, not necrotic. Doxorubicin showed a typical apoptotic pattern after treatment for 24 and 48 h. On the other hand, caspase 3 activation was higher with P2Et fraction than with doxorubicin after treatment for 48 h. Caspase 3 activation was estimated by the cleavage of Ac-DEVD-pNA substrate compared with ethanol treatment (Figure 1c). Finally, DNA fragmentation was assessed and apoptotic body formation was detected after DAPI staining, a distinctive cell death characteristic after DNA fragmentation (Figure 2a). These results confirm that P2Et fraction induces apoptosis of tumor cells through the mitochondrial pathway.

### 4T1 tumor cell line clonogenic capacity is decreased by P2Et fraction

Clonogenic assays are regarded as the gold standard technique with which to determine antitumor activity of fractions and/or isolated compounds [21]. Doxorubicin and P2Et fraction significantly decreased 4T1 cells’ clonogenic ability compared with the negative control (Figure 2b). We may expect that if P2Et fraction has such an effect *in vitro*, the fraction effect *in vivo* could be foreseen to reduce metastasis.

### P2Et fraction induces 4T1 cells to secrete IL-6 and monocyte chemoattractant protein-1 *in vitro*

**Figure 3:**
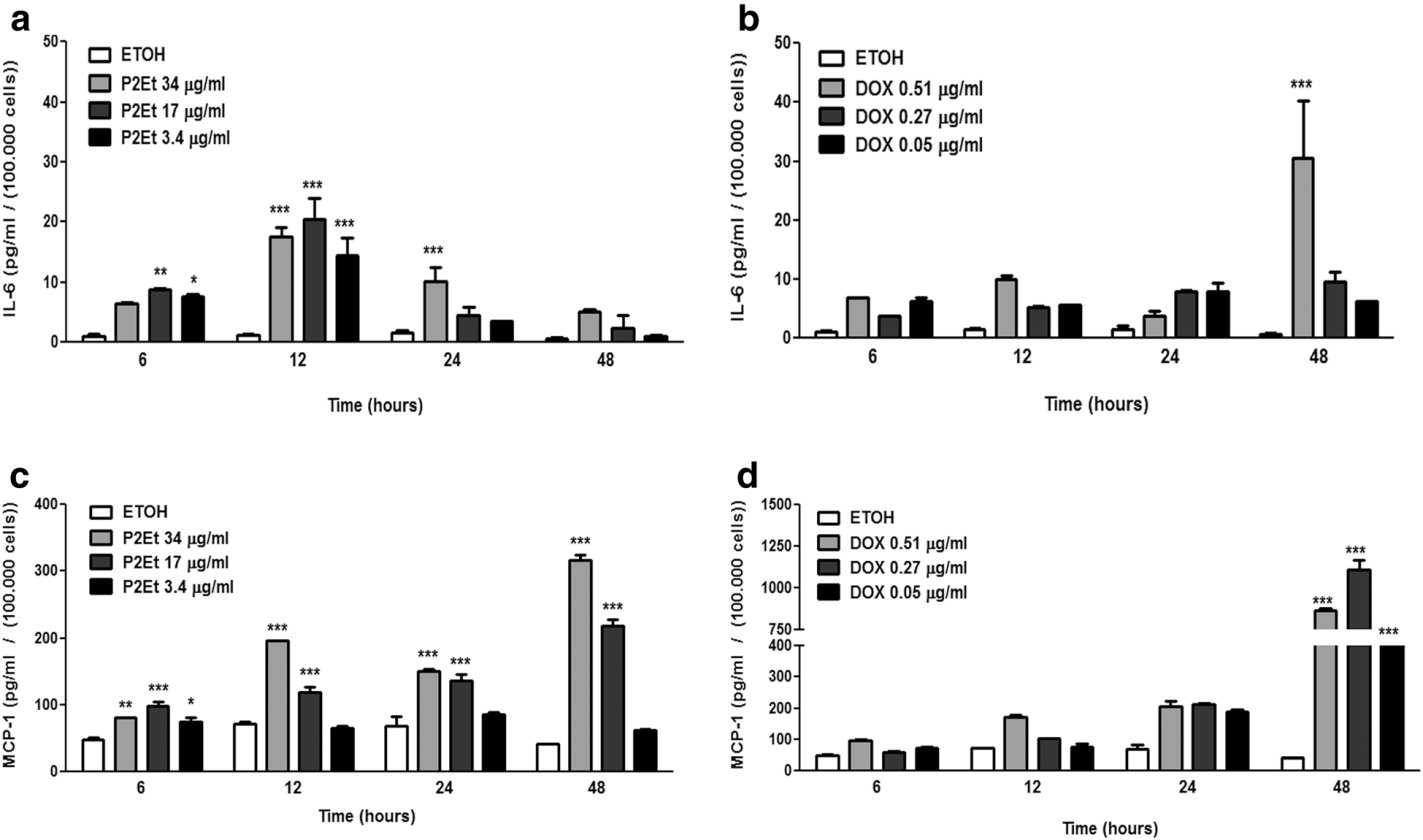
**IL-6 and MCP-1 production by 4T1 cells after treatment with P2Et fraction *in vitro.*** 4T1 cells were treated with P2Et fraction and doxorubicin for 6, 12, 24, or 48 h, and IL-6 (**a**, **b**) or MCP-1 (**c**, **d**) Secretion was determined by flow cytometry (***p < 0.001) versus control.

It is well known that multiple cell components and the tumor microenvironment influence breast cancer progression. Cytokines such as monocyte chemoattractant protein-1 (MCP-1), IL-6, and matrix metalloproteinases have been suggested to promote cancer progression [22]. We evaluated the secretion of IL-6, MCP-1, IL-10, IFN-γ, IL-12p70, and TNF-α by 4T1 cells after treatment with P2Et fraction, doxorubicin, or ethanol (negative control) for 6, 12, 24, and 48 h. Cell culture supernatants were used to determined cytokine secretion by flow cytometry. In basal conditions or after treatment, no significant changes were found in IL-10, IFN-γ, IL-12p70, or TNF-α levels (data not shown). However, IL-6 levels were detected in cells treated with P2Et fraction compared with the control (ethanol) after 6 h of treatment. After 12 h of treatment, IL-6 levels peaked followed by a decrease, and low levels were maintained until 48 h at all P2Et fraction concentrations. Doxorubicin-treated cells showed no significant increase in IL-6 levels at 6 to 24 h of treatment, but the secretion levels peaked at 48 h at a concentration corresponding with the IC50 (Figure 3a, 3b). P2Et fraction and doxorubicin can induce a slight increase in MCP-1 secretion by 4T1 cells in a dose-dependent manner with a peak at 48 h (Figure 3c, 3d). In addition to having a cytotoxic effect on 4T1 cells, P2Et fraction and doxorubicin may also positively modulate IL-6 and MCP-1 secretion, but with different kinetics. Doxorubicin increased IL-6 secretion at the IC50 concentration; while P2Et fraction increased IL-6 secretion at all tested concentrations.

### P2Et fraction inhibits 4T1 cell growth and metastasis in BALB/c mice

**Figure 4:**
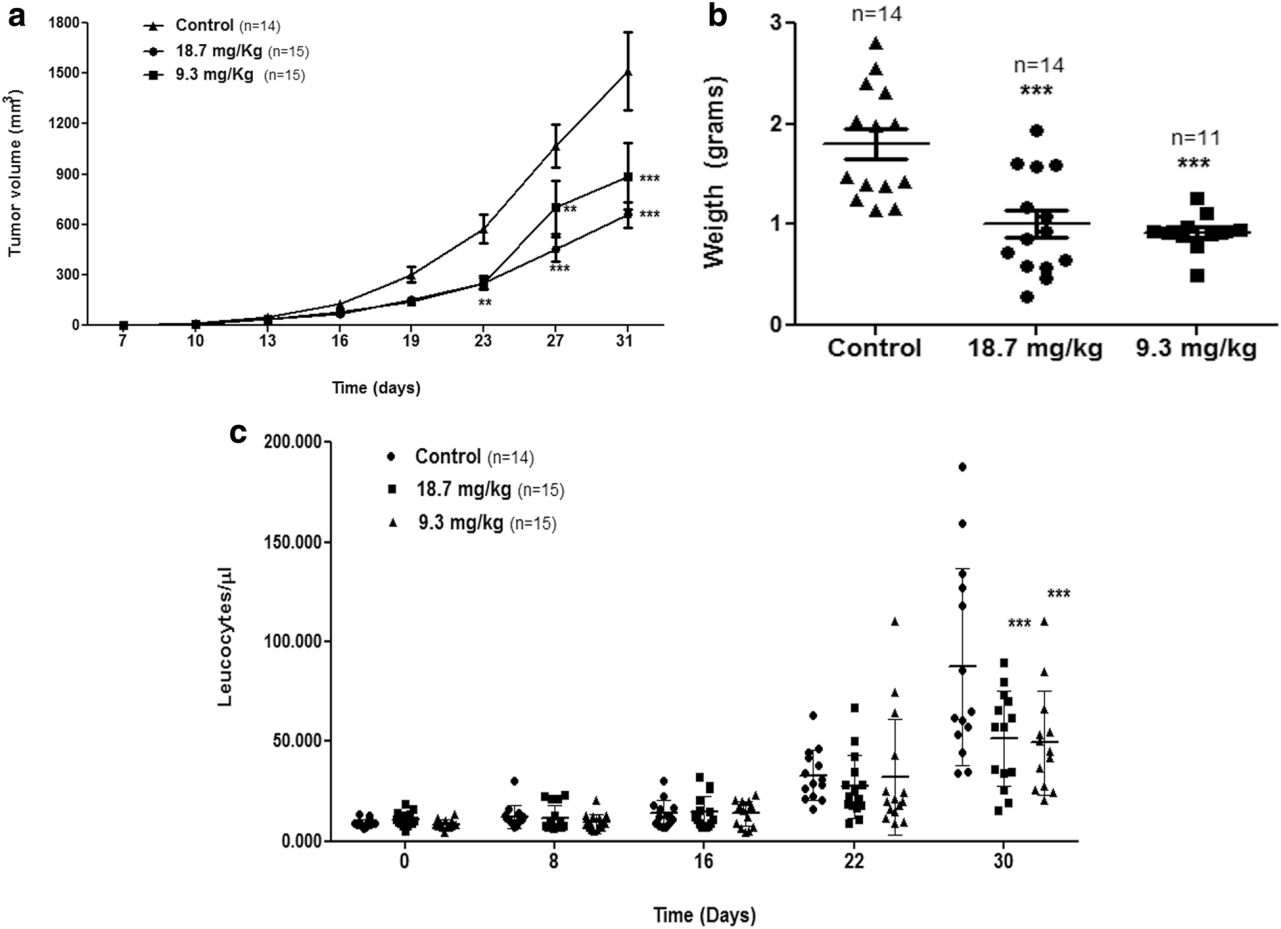
**Tumor growth inhibition and decrease in leukemoid reaction in BALB/C mice by P2Et fraction.** BALB/c mice implanted SC with 1 × 10^4^ 4T1 cells for 5 days and randomly divided into three groups. Group 1 was treated with PBS (vehicle), group 2 was treated with 18.7 mg/kg of P2Et fraction, and group 3 was treated with 9.3 mg/kg of P2Et fraction. Leukocyte count was determined weekly using a hemocytometer (ABX Micros 60). After 31 days, all animals were sacrificed. (**a**) Tumor volume. The graph represents the mean of each group with 7–8 animals per group. (**b**) Tumor weight. The graph represents the total animals by each group. For these experiments we used 7–8 animals per group and each experiment was performed independently two times. (**c**) Leukocyte count. The graph represents the total number animals used per group. For these experiments we used 7–8 animals per group and each experiment was performed independently two times.

To assess the chemotherapeutic effect of P2Et fraction under physiological conditions, a murine model of metastatic breast cancer was used after transferring 4T1 cells into mouse mammary fat pad. The P2Et therapeutic dose was determined following LD50 estimation as described in the Materials and Methods. The LD50 corresponded to 69.6 mg/kg, and the therapeutic dose was 4-fold less to ensure low toxicity (Additional file 2: Figure S2). Female BALB/c mice in groups of 7 to 8 were inoculated with SC injection of 1 × 10^4^ 4T1 cells. After 5 days, the tumors were palpable, and the mice were treated with 18.7 and 9.3 mg/kg of P2Et fraction or vehicle (PBS) twice a week. Figure 4a shows that P2Et fraction significantly reduced tumor growth compared with the control, becoming increasingly marked from day 23 until 30. Similarly, there was a significant decrease in primary tumor weight at the two tested concentrations (Figure 4b).

Inoculation of 4T1 cells into mouse mammary fat pad induces extramedular hematopoiesis, generating the so-called leukemoid reaction [23], a condition also observed in advanced human cancers. Leukocytosis of >50,000 cells/μL is associated with an increase in tumor invasive capacity and size, which is probably caused by tumor cell secretion of GM-CSF [24]. Because the leukemoid reaction in several human cancers is associated with a poor prognosis [25], we evaluated whether P2Et fraction therapy influences the leukocyte number in the peripheral blood of mice. The decrease in leukocyte number, seen since day 22, was considered significant on day 30 (Figure 4c). The latter results suggest that P2Et fraction activity may decrease mediators triggering the leukemoid reaction in this model, which can be beneficial for the disease outcome.

**Figure 5:**
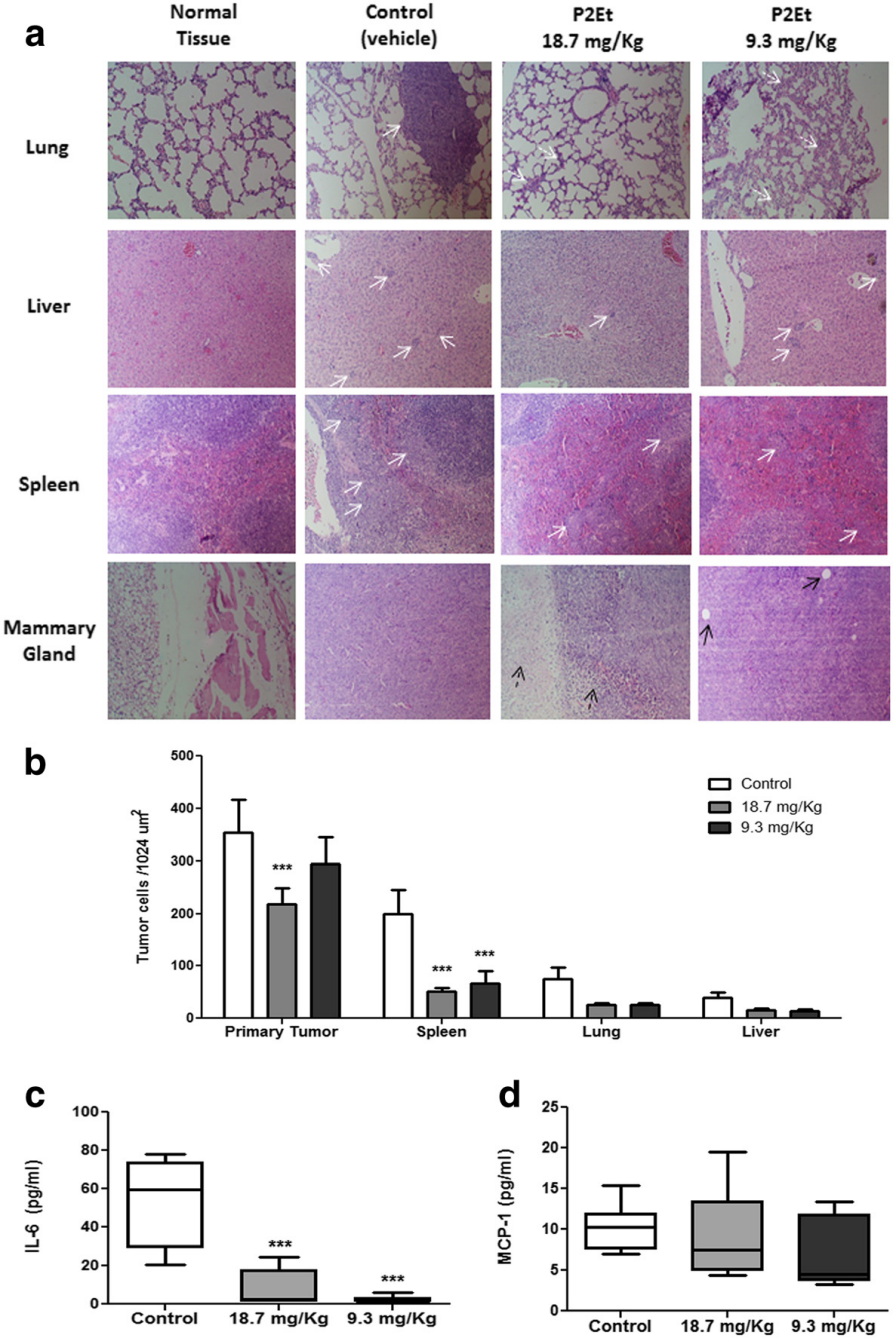
**Reduction of spleen metastasis and decreased IL-6 serum levels in BALB/c mice treated with P2Et fraction.** After treatment, primary tumors and lung, liver, and spleen tissues were dissected and fixed in 10% formaldehyde, embedded in paraffin, and stained with H&E. Metastatic infiltrations were evaluated in each tissue and counted using a microscope with a micrometric grid (magnification power, 10×). (**a**) Tissue Images. White arrows show metastatic tumor infiltration, white dotted arrows show alveolar septum, black arrows show adipocytes, and black dotted arrows show necrotic areas. (**b**) Graphic shows tissue cell number in each (***p < 0.001) versus control. The graphs represents mean of each group with 7–8 animals by group and each experiment was performed independently two times. (**c**) After treatment, mice serum was collected by cardiac puncture, and cytokine estimation was performed using a CBA mouse inflammation kit. (**c**) IL-6 secretion. (**d**) MCP-1 secretion (***p < 0.001) versus control. The graphs represents mean of each group with 7–8 animals by group and each experiment was performed independently two times.

Since the experimental model developed spontaneous metastasis, histopathological analysis to evaluate the number of tumor cells present in lung, liver, spleen, and primary tumor tissues was carried out. Treated mice tissues had fewer tumor cells than did untreated mice tissues (Figure 5a, Additional file 3: Figure S3), with significant differences in the primary tumor and spleen (Figure 5b). Similarly, no tumor cells were found in the bone marrow or brain tissues of either group (data not shown). P2Et fraction exerted activity on the primary tumor as evidenced by the decrease in size and weight of the tumor and the decrease in metastases in the spleen and liver.

### P2Et fraction reduces IL-6 serum levels in BALB/c mice

Microenvironmental factors such as proinflammatory cytokines and adhesion molecules may support tumor progression toward a malignant phenotype by favoring a suitable environment and interactions with tumor cells [26]. As mentioned above, cytokines such as MCP-1 and IL-6 may promote breast cancer progression [22]. There are also reports associating IL-6 with growth and progression of ovarian and myeloma cancers [27,28]. Thus, we quantified IL-6, MCP-1, and the proinflammatory cytokines IL-10, IFN-γ, IL-12p70, and TNF-α in treated and control mice serum. Treated mice had significantly lower IL-6 serum levels compared with the controls (Figure 5c). We also observed a decreasing trend in MCP-1, although it was not significant (Figure 5d). The other tested cytokines showed no differences or were not detected in the serum of mice (data not shown). These results suggest that P2Et fraction diminishes the host inflammatory response, apparently indirectly, and that this change is associated with a decrease in tumor diameter and metastasis control, confirming its potential antitumor function.

**Figure 6:**
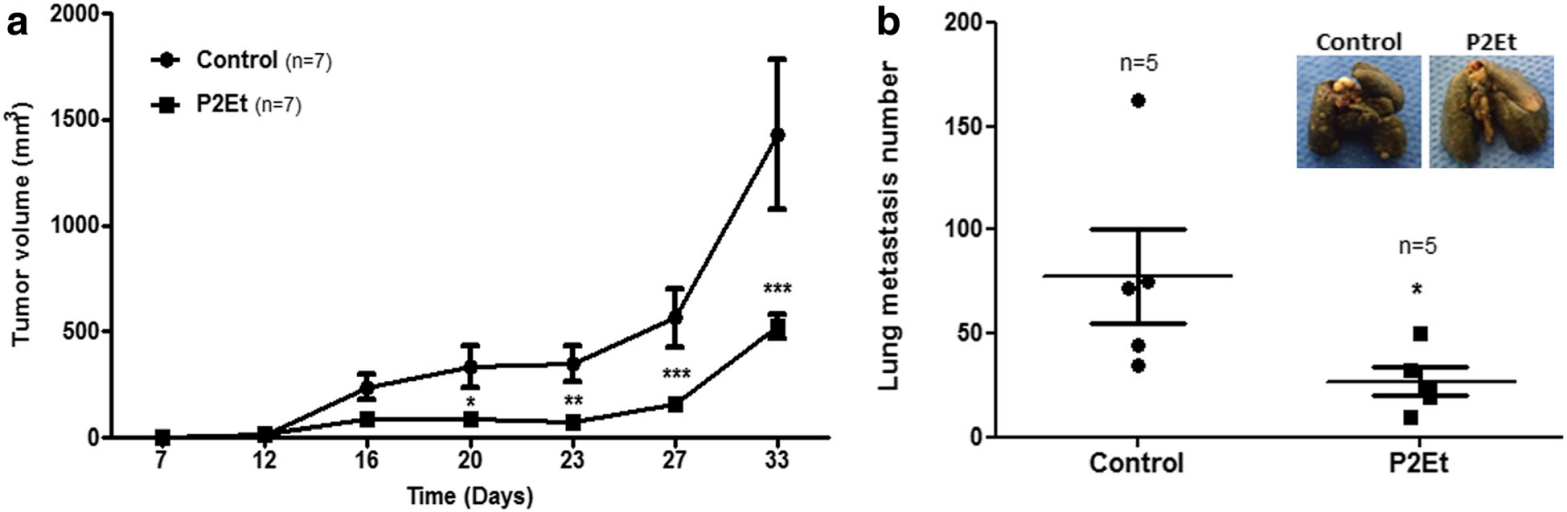
**Tumor growth and lung metastases formation is decreased after treatment with P2Et fraction.** BALB/c mice were randomly assigned in control or treatment groups (7 mice per group). One week prior to tumor cell inoculation (day 0), the treatment group started P2Et fraction consumption administered in water. 4T1 cells (1 × 10^4^) were injected into the right mammary fat pad SC on day 7. After 33 days, all animals were sacrificed. (**a**) Tumor volume. The graph represents the mean of each group with 7 animals per group. (**b**) Lung metastatic number. White tumor nodules against a blue lung background were counted by two independent observers. The graph represents the total animals by each group. For these experiments we used 5 animals per group.

To determine its biological activity and bioavailability, the P2Et fraction was orally administrated to female BALB/c transplanted with 4T1 cells as described in detail in the Material and Methods section. We demonstrated that mice receiving the P2Et fraction had significantly reduced tumor volumes as compared to control treated mice (Figure 6a). In addition, we demonstrated that mice receiving the P2Et fraction exhibited significantly less lung macro-metastasis as judged by India ink staining (Figure 6b).

## Discussion

P2Et is a *C. spinosa*-derived fraction mainly composed of hydrolyzable tannins, gallotannins, and minor concentrations of pentagalloylglucose (PGG), as previously stated [12]. Our study gives evidence of the fraction’s cytotoxic effects on 4T1 cells, inducing apoptosis by the mitochondrial pathway, a path selective for cells with high proliferative rates when compared with normal mononuclear cells or human fibroblasts. Apoptosis is a type of programmed cell death that can be activated by two pathways: extrinsic and intrinsic. The latter involves mitochondrial translocation of pro-apoptotic members of the Bcl-2 family, such as Bax, causing MMP loss followed by the release of several apoptosis mediators, including cytochrome c, into the cytosol [29], which binds Apaf-1 and, in association with other proteins, the apoptosome is formed. This in turn recruits and activates pro-caspase 9, leading to the activation of effector caspases such as caspase 3. Finally, DNA endonucleases are activated [30,31]. This cell death pathway involving caspase activation produces typical morphological changes such as chromatin condensation, nucleosomal DNA fragmentation, nuclear membrane disruption, PS externalization, and formation of apoptotic bodies [29]. We herein demonstrated that P2Et fraction activity induces apoptosis with MMP loss, PS externalization, caspase 3 activation, and DNA fragmentation.

The activities of isolated compounds that are also present in P2Et fraction have been previously studied. Gallotannins or gallic acid derivatives, specifically alkyl gallates and gallamides, have proven cytotoxic effects on L1210 leukemia cells in a dose-dependent manner [32]. *Eugenia jambos* L. (Myrtaceae) plant extract exerts cytotoxic activity on HL60 leukemia cells. This extract contains hydrolyzable tannins (1-O-galloyl castalagin, casuarinin, and gallotannins) and induces apoptosis and DNA fragmentation [8]. Similarly, we previously reported that P2Et fraction has a cytotoxic effect on leukemia cells (K562), inducing apoptosis [12]. The compound’s broad activity range can also be seen in *Terminalia chebula* fruit extract, rich in gallotannins capable of inducing apoptosis in the murine and human breast cancer cells MCF-7 and S115, respectively, among other activities [9].

PGG is a compound also present in P2Et fraction, and it is capable of inducing G1-phase arrest [33], suppressing the phosphorylation and protein level of estrogen receptor alpha by promoting degradation in the lysosome and negatively modulating the ErbB/PI3K/Akt pathway in MCF-7 cells [34]. This causes the cells to become refractory to estrogenic stimulation. In our previous studies, we also observed a G1-phase arrest in MCF-7 cells [12], but not in 4T1 cells, suggesting a cell line-selective mechanism. We also showed that the activities of gallotannins and PGG are regulated by the same cell diversity.

*Caesalpinia spinosa* fruits are well known to be good tannins sources, yet evidence of their cytotoxic or antitumor activity is quite limited. Gali-Muhtasib’s group reported that *C. spinosa* hydrolyzable tannins such as TA lower skin cancer biochemical marker levels, delaying tumor development in mice [13,14]. Similarly, epigallocatechin 3-gallate (EGG), a gallic acid condensed tannin present in green tea, is capable of inducing apoptosis through the mitochondrial pathway in murine breast cancer cells (4T1) [35]. In general, such compounds exhibit low toxicity to normal cells, as observed herein and previously demonstrated for 1-O-galloyl castalagin, casuarinin, and gallotannins from *Eugenia jambos,* which are slightly cytotoxic to human lymphocytes [6].

Our study evaluated the fraction’s ability to modulate tumor cell cytokine production. We found that both P2Et fraction and doxorubicin induced early IL-6 secretion from ER(+) 4T1 cells and in both treatments there was apoptosis induction, inferring that IL-6 favors their latter removal [36]. No significant changes in the levels of the other tested cytokines were found in tumor cell secretion. To control tumor growth *in vivo*, is necessary to induce direct death of tumor cells and regulate microenvironmental and tissue factors involved in primary tumor proliferation and migration. IL-6 is a mediator with dual activity in tumor growth and metastatic processes. Intrinsic production of IL-6 in the ER(+) breast cancer cell lines MCF-7, T47D, ZR-75-1, and SK-BR promotes cell death in response to drugs, inducing morphological changes, and apoptosis with DNA fragmentation [37-39]. On the contrary, in the ER(-) breast cancer cell lines HS578T and MDA-MB-231, IL-6 is related to a drug-resistant phenotype with increased expression of MDR-1 [40]. In our system, IL-6 production by 4T1 cells favored apoptotic cell death.

On the other hand, when overexpressed in organs such as the liver, brain, or lung, IL-6 can promote metastasis by attracting and promoting tumor cells [41]. In patients with breast cancer, IL-6 is associated with a poor prognosis because the levels increase in advanced stages and are associated with a higher number of metastases [42]. We observed a decrease in circulating IL-6 that may have been correlated with the reduction in tumor volume and metastasis; however, it also may have been correlated with negative regulation of the inflammatory microenvironment, favoring the activation of mechanisms implied in tumor elimination.

Additional results suggest that P2Et fraction could modulate different mediators. When 4T1 cells are orthotopically transplanted to BALB/c mice, a leukemoid reaction is induced. This reaction is characterized by leukocytosis of >50,000 cells/μL with predominance of immature granulocytes Gr-1^dim^ and splenomegaly due to massive granulocyte infiltration into the red pulp [23]. P2Et fraction-treated animals showed a reduced number of peripheral blood leukocytes at day 30. The leukemoid reaction has been associated with a poor prognosis in animal cancer models and human cancers [25]. The decrease in response by P2Et fraction therapy could improve the course of the disease.

The *in vivo* P2Et fraction activity showed that the fraction favors not only a decrease in tumor diameter and weight, but also a significant decrease in spleen and liver metastases. 4T1 tumor cells are highly aggressive and metastatic in BALB/c mice, causing death mostly by metastasis and not by the primary tumor [16,43]. Polyphenols, particularly EGG, reportedly have a role in metastasis control in 4T1 models [35] and that their activity is linked to a decrease in proliferating cell nuclear antigen, resulting in a decreased cell proliferation rate.

Tumor progression into a malignant phenotype is favored by different signals generated by microenvironmental factors such as proinflammatory cytokines and adhesion molecules as well as conditions that support the tumor microenvironment by interacting with specific tumor cell receptors [26], promoting breast cancer progression [22]. Thus, high IL-6 serum levels are associated with lower survival, and at later stages, IL-6 can contribute to tumor growth progression [11]. We found that IL-6 serum levels in mice treated with P2Et fraction were significantly lower than those in untreated mice. Similarly, MCP-1 levels were reduced, although not significantly. The latter findings of the decrease in leukemoid reaction and direct activity over tumor cells allow us to conclude that P2Et fraction, besides reducing primary tumor diameter and weight, lessens the migration to distant organs by mechanisms involving the tumor microenvironment as shown by the decreased serum IL6. With regards to data demonstrating that treatment of tumor-bearing mice with P2Et fraction results in significant reduction in serum IL-6, we hypothesize that locally produced IL-6 may improve apoptosis mechanisms and are consumed immediately by tumor cells during its death process thus acting in an autocrine fashion as previously discussed. We concur that tumor reduction after P2Et treatment cannot simply explain IL-6 serum reduction and hypothesize that some other immune-regulatory mechanisms are mediated by the P2Et fraction cannot be excluded, including the inhibition of suppressor macrophages.

Overall, P2Et fraction decreases associated risk factors and primary tumor size, supporting its potential use as an adjuvant in ER(+) breast cancer treatment. Moreover, it is important to note that antitumor activity of the fraction is observed in immunocompetent mice; consequently, tumor microenvironment modulation together with antitumor immune response activation allows for control of distant metastases. We are currently evaluating this hypothesis in our laboratory.

## Conclusions

P2Et fraction exerts its activity on the primary tumor, reduces cell migration to distant organs, and implies the involvement of tumor microenvironment-associated mechanisms in the drop in IL-6 serum levels. Overall, P2Et fraction lessens risk factors associated with tumor progression and diminishes primary tumor size, offering great potential for use as an adjuvant in ER(+) breast cancer treatment.

## Abbreviations

CBA: Cytometric bead array; DAPI: 4,´6-diamidino-2-phenylindole; DMSO: Dimethyl sulfoxide; EGG: Epigallocatechin 3-gallate; H&E: Hematoxylin and eosin; IC50: 50% inhibition cell growth; IP: Intraperitoneally; IL-6: Interleuquin 6; LD50: Lethal dosis 50; MCP1: Monocyte chemoattractan protein-1; MMP: Mitochondria membrane potential; MTT: Methylthiazol tetrazolium; PGG: Pentagalloylglucose; PI: Propidium iodide; PMBC: Human peripheral blood mononuclear cells; PS: Phosphatidylserine; S.C: Subcutaneously

## Competing interests

The authors declare that they have no competing interests.

## Authors’ contributions

The present work was conceived, directed, and coordinated by SF helped by AA. *In vitro* cytotoxicity assays, MMP measurements, Annexin V determinations, DAPI DNA fragmentation testing, cell line maintenance, and *in vivo* experiments were undertaken by CU. Metastatic cell count was carried out by JM. Clonogenic assays were carried out by DC. Plant fraction preparation, characterizations, and statistical analysis were conducted by LP. Cytokine production in serum was carried out by AG. Oral experiments *in vivo* were carried out by JH. All authors read and approved the final manuscript.

## Pre-publication history

The pre-publication history for this paper can be accessed here:

http://www.biomedcentral.com/1472-6882/13/74/prepub

## Supplementary Material

Additional file 1: Figure S1 Dose–response viability curve. 4T1 cells were seeded in 96-well plates and treated with different concentrations of P2Et fraction (250 a 1.9 μg/mL) for 48 h. Viability were determined by the MTT method described in Materials and Methods. The IC50 was calculated using Probit analysis (MINITAB 14.1). Click here for file

Additional file 2: Figure S2 Dose–response curve for acute lethality. Groups of 5 BALB/c mice (6 to 12 weeks of age) were injected IP with P2Et fraction (40, 80, 160 and 320 mg/Kg). Animals were monitored 72 h for mortality and the lethal dose 50 (LD50) was calculated using Probit analysis (MINITAB 14.1). Click here for file

Additional file 3: Figure S3 Reduction of metastasis in BALB/c mice treated with P2Et fraction. After treatment, primary tumors and lung, liver, and spleen tissues were dissected and fixed in 10% formaldehyde, embedded in paraffin, and stained with H&E. Metastatic infiltrations were evaluated in each tissue (magnification power, 100×). White arrows show metastatic tumor infiltration, white dotted arrows show alveolar septum, white circles show aberrant mitotic cells and black arrows show adipocytes. Click here for file
