# Supplementary figures and images for: Gallotannin-rich Caesalpinia spinosa fraction decreases the primary tumor and factors associated with poor prognosis in a murine breast cancer model
Source: BMC Complement Altern Med. 2013 Apr 3;13:74. doi: 10.1186/1472-6882-13-74 (PMC3626639; doi:10.1186/1472-6882-13-74)

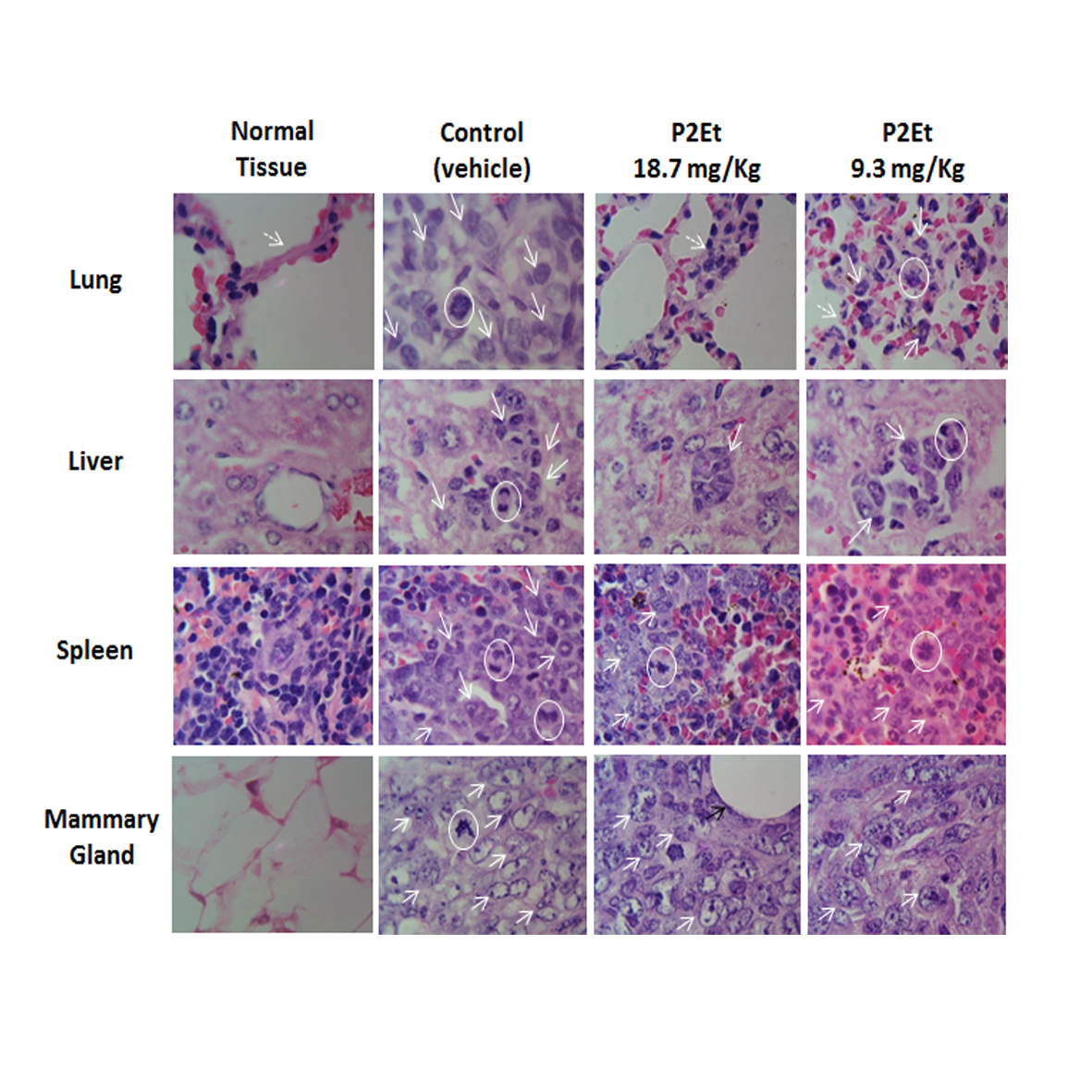

Supplement: Additional file 3: Figure S3 — Reduction of metastasis in BALB/c mice treated with P2Et fraction. After treatment, primary tumors and lung, liver, and spleen tissues were dissected and fixed in 10% formaldehyde, embedded in paraffin, and stained with H&E. Metastatic infiltrations were evaluated in each tissue (magnification power, 100×). White arrows show metastatic tumor infiltration, white dotted arrows show alveolar septum, white circles show aberrant mitotic cells and black arrows show adipocytes. [file 1472-6882-13-74-S3.tiff]
